# Medical Clinic

**Published:** 1883-02

**Authors:** Jas. T. Whittaker

**Affiliations:** Cincinnati; At the Good Samaritan Hospital


For Contents See Page 128

THE

### Independent Practitioner.

VOL. IV. FEBRUARY, 1883. NO. 2.

### Original Communications.

*We are not responsible for the opinions expressed by contributors.*

ARTICLE I.

### MEDICAL CLINIC

BY PROF. JAS. T. WHITTAKER, M. D., CINCINNATI.

At the Good Samaritan Hospital, January 8, 1883.

REPORTED BY DR. W. H. WENNING.

CASE I: *Œdema Pulmonum.*—Mrs. S., aged sixty-three; native of Ireland, resident of this city. Admitted January 5.

States that she took a very severe cold, accompanied by cough and dyspnœa. She has often had a difficulty of breathing whenever she took cold, she said. She also acknowledged to drinking pretty freely at times, but never to drunkenness.

Her temperature when admitted was 100½ degrees; respiration, 48; pulse, 120 to 130. The temperature afterward fell to normal, but the respirations continued very rapid, being about 50 per minute when at rest. The expectoration is muco-purulent.

Auscultation reveals abundant coarse and fine rales of the mucous variety all over the chest. Percussion reveals nothing abnormal.

The urine contains a trace of albumen, but has not yet been subjected to microscopic examination.

*Gentlemen:* After having heard the history of this case by Dr. Paulding, I call your attention to the general appearance of the patient.

You notice the distress depicted on her countenance, the shallowness and rapidity of respiration, and the bluish pallor of the face. You also observe that the decubitus is on the left side; she cannot lie on the right side, and becomes faint when she sits up. Her cough is moist and the expectoration is abundant. From these symptoms you evidently will suspect disease of the lungs, but we must determine what is the exact condition. Our first suspicion is pneumonia. On percussion, however, you hear tympanitis instead of dulness, as you would expect to find in pneumonia, at least in the stage of hepatization; then there is here unusual resonance. On auscultation I hear no bronchial respiration but fine moist rales, especially at the base of both lungs. We must, therefore, exclude pneumonia, at least in the stage of consolidation. You have heard from the history that she has been subject to the delirium potatorum. Such a condition as this is apt to come on frequently and suddenly in these subjects, and the cause of the dyspnœa is often said to be due to the lungs, which, however, are not the cause of the disease. It is more probable that this is a case of chronic Bright's disease. The urine is clear and scant, and contains but a small amount of albumen; but these are often just the worst cases of this disease, namely, cirrhoses of the kidneys. The final catastrophe of cases of this nature is here, namely œdema pulmonum, a not infrequent termination of Bright's disease, and next to brain disease the most serious complication of a cirrhotic kidney. This condition acounts for the difficult respiration. As the fluid accumulates in the lungs it fills the air-cells and the patient struggles for air and is in danger of suffocation. The interest centres now altogether in the condition of the lungs; there is not here the brick-dust or prune-juice expectoration characteristic of pneumonia; the sputum is frothy and purulent, and from the cough as you just hear it you recognize that the secretion in the tubes is in great quantity.

But you may also find this condition in chronic valvular disease, which we eliminate here, however, by the absence of any murmur. Some of these cases of heart disease die of manifestations on the part of the brain when the aortic valves are the seat of disease, but in the most frequent if not dangerous form, namely, mitral regurgitant lesion, we may observe œdema of the lungs.

The subjective signs we have just been considering. Besides these we have plain objective signs, as want of expansion of the chest and increase of resonance, due to the contraction of the muscles of the chest. On auscultation we discover that the fine crepitant rale so characteristic, though by no means pathognomic, of pneumonia is not here, but fine mucous rales everywhere, indicative of the fluid in the air-cells.

The dyspnœa and partial cyanosis are due to this occlusion of the air-cells, but patients with Bright's disease often show dyspnœa independently of œdema pulmonum, as the effect of uræmic poisoning. In fact most of these attacks are uræmic asthmas and not mere mechanical occlusions, and the dyspnœa is only one of the numerous brain symptoms of Bright's disease.

The prognosis of œdema pulmonum, from whatever cause, is always grave. Though not every one must necessarily terminate fatally, it is an indication of extreme danger.

For this reason stimulation is called for. Digitalis and alcohol must be freely given. We appeal directly to the heart and indirectly to the kidneys by the administration of digitalis, which by stimulating the heart forces more blood through the kidneys, and consequently more urine is excreted. Our patient takes a tablespoonful of the infusion of the leaves every two hours and two tablespoonfuls of whiskey-punch every three hours. I repeat it, digitalis pushes the blood through the lungs and whips up the action of the kidneys by propeling a larger amount of blood through them. In these cases it often acts like a charm, and in many cases which have been neglected and death seems imminent from œdema of the lungs, the prompt administration of digitalis produces a different aspect in the cases in eight or ten hours. This patient has improved greatly since this morning. You may give the fluid extract or the tincture, but the best preparation by far is the infusion of the leaves, freshly made. To many cases of Bright's disease gone so far as to be bloated with dropsy, you may give months or even years of life with digitalis. Combine it in these cases with a salt of soda or potash and you will get a quicker effect. But there is no question of curing this patient, I may say, now that she is removed. Should she recover from this attack, she will succumb later to some other lesion peculiar to, and apt to occur in, this form of kidney disease. I show you the case because of the general interest connected with œdema pulmonum, so often in the long chain of disease processes belonging to cirrhosis of the kidneys, the final link.

[The patient died four days later, but an autopsy was not permitted.]

Case II: *Typhoid Fever with Relapses.*—Brother Modesta, aged twentyeight, a native of Ohio, resident of Notre Dame University; occupation, teacher; admitted November 26, 1882. Patient states that he had been sick for a week, but had not taken to his bed, thinking that it was some transient ailment that would soon pass away.

He had epistaxis at the start, chilly sensations, fever and diarrhœa. When admitted his temperature was 102½ degrees in the evening and 101½ degrees in the morning, which record was maintained without any material deviation until the fifteenth day of his illness, when there was a slight decline and the temperature went down about one degree per day until it was normal. The axillary temperature alone was taken.

The patient after a day or two was allowed more substantial food, and upon the third day sat up for a half hour while his bed was being changed (December 6th).

On the fourth day after his temperature reached normal there was another rise and the patient complained of severe paroxysms of pain in the rectum, which were relieved by injections of hot water. The temperature continued high for three days, then fell to normal, to again rise gradually for a few days until it reached 101½ degrees in the mouth and continued at that height with the exception of morning remissions for three weeks. The rise and fall marked the typical typhoid curve.

Again for the fourth time the temperature rose, continued a week and fell by lysis. His food throughout has been fluid—milk, soup, beef tea, egg nogg, etc. There has been no diarrhœa, but very little cough and that not constant. No abdominal distension and no head symptoms. The patient has used the bed pan and taken exceptional care of himself. The last rise of temperature seemed to be controlled by quinine, which had no effect on previous occasions.

The history of this case shows that we have had to deal with a mild typhoid fever, but with several distinct relapses. Although this patient was surrounded with all additional precautions on account of the death from perforation that occurred in a mild case here a short time ago, he had three, if not four distinct relapses, and though each attack was mild, the sum of them all has reduced the patient to an extreme degree.

Relapses occur in about eight per cent. of all cases of typhoid fever. They are, as a rule, milder than the original attack, and when a patient succumbs in such relapses it is because he is left more debilitated by the original attack than before the onset of the disease. Death may thus occur independently of complications in the mildest as well as in the most severe forms of typhoid fever.

The history further shows that the fever in this case was abortive, terminating in fifteen days. Now not very rarely the disease is cut off at the end of the second week instead of the third week, and if a remedy shall have been administered at the time, it is credited with having cured the disease. This patient had a normal temperature for two or three days, when he was allowed some raw meat. A relapse then occurred, which I do not, however, attribute to the diet, or to the fact that the patient sat up in bed, but to the original poison. It was formerly supposed that with every renewed attack there was also introduced some new poison into the system. This is not so; a relapse means that the contamination is still present in the body and its action on the system has been delayed. If a patient has had typhoid fever once, he has had the disease, as a rule, once for all for the rest of his life. If you put organic substances in different test tubes at the same time exposed under the same conditions to the same atmosphere, you will find that putrefaction occurs on different days in the different tubes. So also here Peyerian glands may be successively attacked, and relapses occur independently of indiscretions of any kind.

A matter of consolation for the physician as well as the patient is the fact that the relapse is always milder than the first attack. Generally the patient will nevertheless recover unless too much reduced by the original disease.

A case of this kind might at first be mistaken for relapsing fever. This disease is rare here, but occurs frequently in sea-port towns. In Philadelphia it was not long ago epidemic. It somewhat resembles typhoid fever, but does not show its step-ladder rise of temperature in the first week. In relapsing fever the patient has a temperature at the start of about 103 or 104 degrees, when he is ill for a week with continued fever, but at the end of this time or nine days the temperature falls suddenly, to remain normal for a week, when it takes again a new rise. Thus one or several relapses may occur. This is the main point of diagnosis, though the absence of nose bleeding, rose spots and head symptoms in relapsing fever are additional factors. Lastly, relapsing fever shows the characteristic spirochates in the blood during the stage of pyrexia.

In some cases the diagnosis becomes more difficult between typhoid fever and miliary tuberculosis. The nose-bleeding, chill, rise of temperature, bronchial cough, meteorism and diarrhœa may all be present in tuberculosis, and it is only by the examination of the sputum for the bacillus according to the methods you have so often heard described that you can positively establish the diagnosis.

The case before us, however, leaves no room for doubt on account of its typical course. The occurrence of one relapse is not uncommon, but it is unusual to have two or three. This patient is now in a fair way of recovery, but he must not have any solid food for two or three days. Then he may have at first some raw meat or underdone beefsteak, sweet-bread, soft boiled egg, game, and later the more digestible vegetable foods, as celery, baked potatoes, etc. As long as there is any headache or symptoms of prostration he must not rise from his bed. He may take alcohol freely, because it is the best food and drink and drug in every case of fever.

Case III: *Locomotor Ataxia.*—The last case that I present to you today is that of this young man, who, as you see him entering the room, has some difficulty in walking. He says that about one year ago he felt his legs getting weak. He also felt a dizziness in the head, severe pain in the back and cramps in his lower extremities. I ask him to stand still with his eyes shut, and you see that he wavers. It is with the greatest difficulty that he can stand on the left leg alone and it is next to impossible for him to stand on the right alone. I test the patellar tendon reflex and find it good in the right, but very defective, almost *nil*, in the left knee. In testing for this valuable sign you must, however, recollect that there is no rule without exceptions. This phenomenon is found absent normally in about two per cent. of healthy adults and five per cent. of children. In very fat persons it is difficult to elicit it unless they are made to sit on a table, with the legs hanging over its edge. When it has once been recognized as having been present and is then abolished it is a diagnostic sign of the greatest value.

What is the matter with this patient? Locomotor ataxia. You have by this time become perfectly familiar with this disease, for this is the fourth time in as many weeks that I have been able to present this disease. When locomotor ataxia has once progressed so far as in this instance there is no difficulty in recognizing it, but we ought to endeavor to diagnosticate it early.

One early feature of this affection is parasthesia, as a sense of crawling of ants on the limb, a symptom which has here been absent and is only recognized, as are the various alterations in sensation, by those whose attention is easily arrested by anomalies. An individual like this, laboring from day to day, pays little heed to such sensations. Sometimes this prodroma precedes the development of ataetic symptoms, as you see them here, even ten to fifteen years. Disturbances of vision, satyriasis and impotence, and lancinating pains in the pelvis are other pioneer symptoms in this disease. We hope to hold the disease in check for a while at least by such measures as I have mentioned to you before, viz., Faradization, cold shower baths, rest, etc. Absolute rest is of great value, and it is for this reason that these cases can be treated much more successfully in a hospital than in private practice.

Eulenburg maintains that he has cured locomotor ataxia with the salts of silver. The use of silver internally in this disease is not new, but Eulenburg's method, the hypodermic injection of the subsulphate, is new, and is recommended because of the change which the remedy suffers in internal administration.

We may never forget to look for the cause of the disease ; seventeen out of twenty cases have a history of preceding syphilis. This patient had a hard swelling in the groin five or six years ago, which speaks for the character of the disease. This indicates the line of treatment to be followed first. He shall take twenty grains of potassium iodide three times a day, and we are led to expect some benefit from it, because he has never been put upon this treatment before.

---

Effects of Alcohol.—"Dead drunk" is described by savants of the Paris Biological Society to be a condition in which there is a proportion of one part of alcohol to 195 parts of blood in the circulation. Should the proportion ever come to be one part of alcohol to 100 of blood, death would ensue. This might happen, and, in fact, has happened repeatedly, where a very large quantity of alcoholic liquor is swallowed at one time and quickly. In ordinary drinking consciousness is lost, and with it the power to drink more, before the proportion of alcohol in the circulation becomes fatal.

